# Bariatric surgery and subsequent weight loss lead to a reversal of peripheral blood monocytosis in obesity

**DOI:** 10.1038/s41366-026-02093-4

**Published:** 2026-04-24

**Authors:** Marco Krasselt, Kathleen Friedrich, Laurin Braune, Kathrin Rothe, Matthias Blüher, Peter Kovacs, Arne Dietrich, Manuela Rossol, Michael Stumvoll, Ulf Wagner

**Affiliations:** 1 Rheumatology, Medical Clinic III: Endocrinology, Nephrology and Rheumatology, Department of Internal Medicine, Neurology and Dermatology, University of Leipzig Medical Centre, Liebigstr. 20, 04103 Leipzig, Germany; 2 Endocrinology, Medical Clinic III: Endocrinology, Nephrology and Rheumatology, Department of Internal Medicine, Neurology and Dermatology, University of Leipzig Medical Centre, Liebigstr. 20, 04103 Leipzig, Germany; 3 Helmholtz Institute for Metabolic, Obesity and Vascular Research (HI-MAG), Helmholtz Zentrum München at the University of Leipzig and University Hospital Leipzig, Ph.-Rosenthal-Str. 27, 04103 Leipzig, Germany; 4 German Center for Diabetes Research (DZD), Ingolstädter Landstraße 1, 85764 Neuherberg, Germany; 5 Department of Visceral, Transplant, Thoracic and Vascular Surgery, University of Leipzig Medical Centre, Liebigstr. 20, 04103 Leipzig, Germany; 6 Molecular Immunology, Brandenburg University of Technology, Universitätsplatz 1, 01968 Senftenberg, Germany; 7 LeiCeM - Leipzig Center of Metabolism, Leipzig University, Leipzig, Germany

**Keywords:** Obesity, Obesity, Translational research

## Abstract

**Objective:**

To examine changes in monocyte subpopulations and surface markers in people with obesity before and after bariatric surgery, and their relation to weight loss, inflammation markers, and comorbidities.

**Methods:**

Peripheral blood mononuclear cells (PBMCs) were isolated from patients with obesity designated for bariatric surgery at three different time points: before surgery (=baseline), six months and twelve months after the intervention. PBMCs were analyzed using flow cytometry to distinguish the different monocytic subpopulations. At each visit, anthropometric measures and routine laboratory parameters (e.g., C-reactive protein) have been determined.

**Results:**

111 individuals with obesity (59.5% female, mean age 45.2±11.3 years) with a median body mass index (BMI) of 48.4 kg/m^2^ were included into this study. Median weight loss was 44.5 kg. The absolute monocyte count decreased significantly after surgery within twelve months (*p* = 0.0035). Classical monocytes, non-classical monocytes, intermediate monocytes, and monocytic myeloid-derived suppressor cells (M-MDSC) decreased significantly after the surgical intervention within six to twelve months. CD14^bright^/CD56^+^ monocytes did not change significantly during twelve months of observation. Surface expression of CD14 increased in both classical and intermediate monocytes (*p* = 0.0272 and 0.0087, respectively) within 6 months whereas CD16 declined across all monocyte subpopulations at every time point. The total monocyte counts as well as numbers of non-classical monocytes were significantly higher in patients with obesity and type 2 diabetes mellitus. COVID-19 containment measures resulted in a longitudinal reduction in the number of patient evaluations.

**Conclusions:**

Following bariatric surgery and the resulting weight loss, the obesity-associated perturbation of the monocyte compartment was largely reversed. Normalization of both the total monocyte pool and of monocyte subpopulations, particularly those with pro-inflammatory properties such as intermediate monocytes, could contribute to a risk reduction of known co-morbidities of obesity such as chronic inflammation, impaired glucose regulation, and an increased risk of cancer.

## Introduction

Obesity is associated with several comorbid diseases and conditions including impaired glucose tolerance, fatty liver disease, coronary artery disease and some types of cancer [1]. Some of these conditions (e.g. type 2 diabetes mellitus, T2DM) are at least in part the consequence of a chronic tissue inflammation triggered by macrophages that accumulate in adipose tissue (adipose tissue macrophages, ATM) and release inflammatory mediators [2]. Importantly, the NLR family pyrin domain containing 3 (NLRP3) inflammasome seems to be involved in this process. It has been demonstrated that obesity is related to the assembly and activation of the NLRP3 inflammasome and caspase 1 cleavage, ultimately resulting in the maturation of the proinflammatory cytokines IL-1β and IL-18 [3].

Peripheral blood monocytes differentiate into macrophages after immigration into the tissue and are a major source of macrophages in adipose tissue in particular [4–7]. The attraction of monocytes to adipose tissue is mediated by the monocyte chemoattractant protein-1 (MCP-1) and its receptor C-C chemokine receptor type 2 (CCR2) [5, 8]. Peripheral blood monocytes are increased in people with obesity [7, 9], and immigrate into adipose tissue in increased numbers, and this effect is most prominent in visceral adipose tissue [10]. Monocytes are not a homogenous population, but consist of three major subpopulations: classical monocytes (CD14^bright^/CD16^-^), intermediate monocytes (CD14^bright^/CD16^+^) and non-classical monocytes (CD14^dim^ /CD16 + ) [11]. Of particular interest are intermediate monocytes: This subpopulation is associated with several inflammatory rheumatic diseases, ageing and coronary artery disease (CAD) [12–28].

The classical monocytes harbor another small subpopulation, the CD14^bright^/CD56^+^ monocytes which are expanded in autoimmune diseases such as rheumatoid arthritis (RA) and Crohn’s disease. They produce more reactive oxygen intermediates and pro-inflammatory cytokines in RA and are more efficient antigen-presenting cells [29, 30].

Investigating the peripheral monocyte compartment in obesity, we were able to show that it is altered in patients with a BMI above 27 kg/m^2^ [31]. Compared to lean individuals, obesity is characterized by increased numbers of total monocytes, classical monocytes, intermediate monocytes, CD14^bright^/CD56^+^ monocytes, and monocytic myeloid-derived suppressor cells (M-MDSC) [31]. This monocytosis positively correlates to clinical and laboratory markers of obesity such as body mass index, waist circumference, triglycerides, CRP, and HbA1c. The monocytic compartment seems therefore biased towards proinflammatory and immunosuppressive phenotypes, potentially contributing to a chronic low-level systemic inflammation termed metaflammation, and possible cancer development [31].

We hypothesized that bariatric surgery might normalize the monocyte compartment in individuals with obesity, since such procedures and the resulting loss of weight are known to reduce inflammation and induce remission of T2DM [32].

The aim of this study was, therefore, to investigate longitudinally the described alteration in the monocyte compartment of people with obesity before and after bariatric surgery, to better understand the impact of obesity on innate immunity.

## Methods and study design

### Study participants

In collaboration with the Integrated Research and Treatment Center (IFB) Adiposity Diseases of the Medical Faculty of Leipzig University, adult individuals with obesity were consecutively recruited between July 1, 2019 and June 30, 2020, if they had been scheduled for bariatric surgery (sleeve gastrectomy, Roux-en-Y gastric bypass surgery, omega loop gastric bypass). Patients underwent blood sampling and longitudinal analysis of peripheral monocyte phenotype, absolute monocyte count and further investigation of clinical as well as laboratory parameters. Obesity classification was based on the definition of the World Health Organization (WHO), i.e. body mass index [BMI] ≥30 kg/m^2^. Blood samples were obtained longitudinally at bariatric surgery (baseline) and both, six and twelve months thereafter.

Exclusion criteria were pregnancy, chronic inflammatory diseases, known infection during the last four weeks, hypoproteinemia, renal insufficiency (glomerular filtration rate ≤30 ml/min/1.73 m^2^), leukopenia (≤3 per μl), and known malignancies.

Blood samples for PBMC-isolation and clinical characteristics (Table 1) were taken in the fasted state. To assess body composition of the participants, single frequency bioelectrical impedance analysis (SF-BIA) was used. The whole-body impedance measurement technique hand-to-foot (HF-BIA) was conducted and analyzed with BodyComposition V 9.0 Professional (Software BodyComposition, MEDI Cal HealthCare, Karlsruhe, Germany).

**Table 1:** Clinical and laboratory characteristics of the study patients at baseline and 6 and 12 months after surgery. Shown are numbers (%), mean with standard deviation (±SD) or median with interquartile range (IQR).

Characteristics	Baseline	6 months	12 months
**n (%)**	111	46	16
**female, n (%)**	66 (59.5)	27 (58.7)	10 (55.6)
**age, mean [years]**	45.2±11.3	46.9±10.5	51.5±15.2
**BMI, median [kg/m**^**2**^**]**	48.4 (12.2)	37.3 (10.1)	34.2 (12.8)
**body fat, mean [kg]**	76.1±24.3	44.7±22.3	34.4±9
**T2DM, n (%)**	55 (49.5)	19 (41.3)	8 (42.1)
**CRP, median [mg/dL]**	8.1 (10.7)	2.5 (5.0)	1.6 (3.24)
**LDL/HDL ratio**	2.6 (1.3)	2.3 (1.3)	1.9 (1.6)

*BMI* body mass index, *CRP* C-reactive protein, *T2DM* Type 2 Diabetes mellitus.

The design of the study was approved by the ethics committee of the University of Leipzig (AZ 044-16-ff) and written informed consent was obtained from all participants before study enrolment.

### Materials

Flow cytometry antibodies fluorescein isothiocyanate (FITC)-conjugated anti-CD14 (clone TÜK4), phycoerythrin (PE)-conjugated anti-CD16 (clone REA423), allophycocyanin (APC)-conjugated anti-CD56 (clone REA196) and APC-conjugated anti-HLA-DR (clone AC122) and appropriate isotype controls were obtained from Miltenyi Biotec, Bergisch Gladbach, Germany.

### PBMC Isolation

Human PBMCs were isolated using Ficoll-Paque (GE Healthcare Life Sciences, Chicago, IL, USA) density gradient centrifugation and washing in EDTA-containing PBS.

### Flow cytometry

PBMCs (1 × 10^6^/100 μl) were stained with CD14-FITC, CD16-PE, CD56-APC antibodies, and anti-HLA-DR-APC antibody and isotype controls for 20 min at 4° C. Cells were washed twice with PBS supplemented with 2% FCS and 0.1% sodium azide and fixed with 3% formaldehyde. Samples were measured using the BD LSR II and analyzed with FlowJo Version 8.7 (Tree Star) software. Gating strategies to identify the different monocyte subpopulations have been further discussed and shown before [31]. Monocyte subpopulation numbers were calculated as follows: (absolute monocyte number ∗ percentage of monocyte subpopulation)/100.

Determination of clinical laboratory variables was performed at the Institute of Laboratory Medicine, Clinical Chemistry and Molecular Diagnostics, University of Leipzig. Measurement of C-reactive protein (CRP), HbA1c, cholesterol, LDL-/HDL-cholesterol, as well as triglycerides, was performed according to manufacturer’s protocol on an automated laboratory analyzer Cobas 8000 (Roche Diagnostics, Mannheim, Germany). Absolute leukocyte and monocyte numbers were determined according to manufacturer’s protocol on an automated laboratory analyzer XN-9000 (Sysmex, Norderstedt, Germany).

### Biostatistical analysis

To describe continuous data, mean and standard deviation (SD) or median and interquartile range (IQR) were used as appropriate. Categorical data were described with absolute and/or relative frequencies. To compare the frequencies of categorical variables, fisher’s exact test was performed. For comparison of continuous data, student’s *t* test or Mann-Whitney U, as appropriate, was used. To complement group comparisons, a paired t test between all patients that were enrolled and completed month twelve visit was conducted. To assess the difference of three or more groups, analysis of variance (ANOVA) or Kruskal-Wallis test with correction for multiple comparisons (Tukey) was done. A significant statistical difference was assumed when the *p* value was below 0.05.

All statistical analyses were conducted using GraphPad PRISM Version 10.2.1 for Mac OS (GraphPad Software Inc., San Diego, CA, USA).

## Results

### Study population

**Fig. 1:**
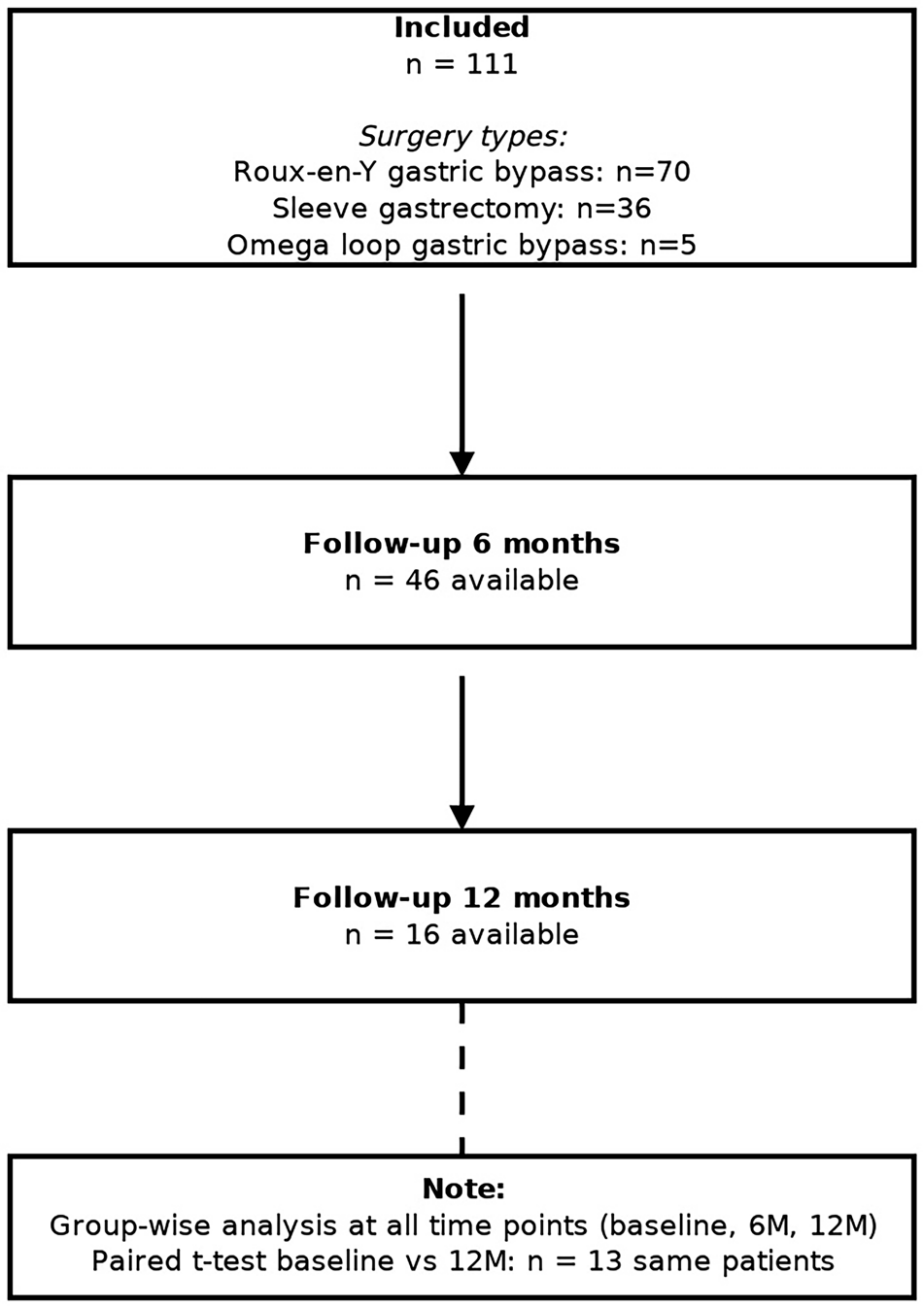
Flow diagram showing patient recruitment. Flow of included patients and available follow-up data at baseline, 6 months, and 12 months; group-wise analyses were performed at each time point, and paired baseline-to-12-month analyses included 13 patients with complete data.

In total, 111 patients with obesity undergoing bariatric surgery (59.5% female, mean age 45.2±11.3 years) have been included into this study. The majority of patients underwent Roux-en-Y gastric bypass (*n* = 70), followed by sleeve gastrectomy (*n* = 36) and omega loop gastric bypass (*n* = 5). The median body mass index (BMI) was 48.4 kg/m^2^ and 49.5% of the analysed individuals had type 2 diabetes mellitus (T2DM) at baseline. Impaired glucose tolerance was seen in approx. 10% (see Table 1 for further details on the patient population and changes in baseline characteristics after six and twelve months). Common compliance challenges in bariatric care, such as poor adherence to follow-up visits, led to substantial patient attrition. These were exacerbated by COVID-19 restrictions and lockdowns, resulting in missed appointments (particularly at month six), rescheduled assessments, and reduced blood sampling. Laboratory capacity for flow cytometry was further limited by staff shortages (Fig. 1).

### Monocyte counts are linked to anthropometric measures and laboratory markers

**Fig. 2:**
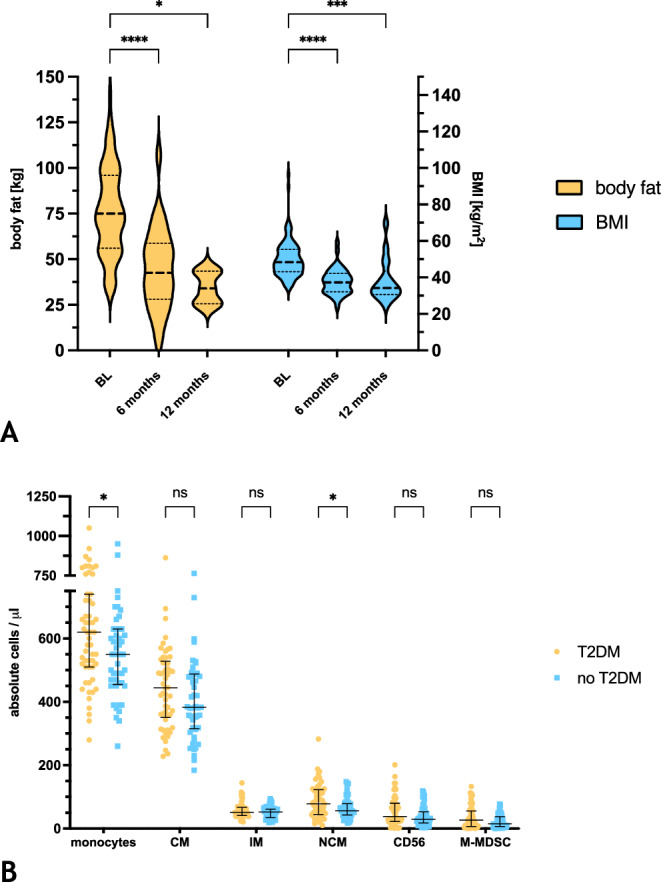
Changes of total body fat (kg) and BMI (kg/m^2^) after bariatric surgery and different distribution of monocyte subpopulations in patients with and without T2DM at baseline. **A** Violin plots showing the changes of body fat proportion and BMI after bariatric surgery. Lines indicate median (dashed line) and interquartile range (IQR, dotted line). **B** Distribution of absolute monocytes and the monocytic subpopulations according to the presence of a known T2DM at baseline. Each dot represents one patient. Shown are median and IQR, each dot represents one patient. Significances as indicated. ^*^ – *p* < 0.05, ^***^ – *p* < 0.001, ^****^ – *p* < 0.0001; *BL* baseline, *BMI* body mass index, *CM* classical monocytes, *IM* intermediate monocytes, *NCM* non-classical monocytes, *M-MDSC* monocytic myeloid-derived suppressor cells, *T2DM* type 2 diabetes mellitus.

Bariatric surgery had a significant impact on both, body fat and BMI (Fig. 2). Body fat decreased from 76.1 to 44.7 kg after six months (*p* < 0.0001) and further to 34.4 kg after 12 months (*p* = 0.0251). Concordantly, BMI changed from 48.4 to 37.3 kg/m^2^ after six (*p* < 0.0001) and 34.2 after 12 months (*p* = 0.0007) (Fig. 2A). At baseline, both overall monocytes as well as non-classical monocytes were significantly higher among participants with T2DM compared to those without diabetes (*p* = 0.03 and 0.04, respectively, Fig. 2B).

Correlations between typical markers of inflammation and glucose/lipid metabolism as well as anthropometric measures and monocyte subpopulations can be obtained from Supplementary Table 1. The overall monocyte count was positively correlated with CRP levels at baseline (*r* = 0.198, *p* = 0.039) and after six months (*r* = 0.394, *p* = 0.007); one year after surgery, only a trend was remaining, most likely due to the lower number of patients (*r* = 0.426, *p* = 0.078, Supplementary Table 1). In addition, BMI was correlated to overall monocytes particularly after twelve months (*r* = 0.526, *p* = 0.025).

Interestingly, non-classical monocytes counts were related to several parameters. CRP values six and twelve months after surgery were not only significantly lowered compared to baseline, but formally also within the normal range given from our central laboratory. Nevertheless, frequencies of non-classical monocytes were found to still correlate stringently with CRP values six and twelve months after surgery (*r* = 0.404, *p* = 0.005; and *r* = 0.688, *p* = 0.013, respectively), indicating those residual CRP levels might still be disease relevant. Surprisingly, body fat was strongly negatively associated with non-classical monocytes after twelve months (*r* = -0.981, *p* = 0.019). Further correlations can be obtained from Supplementary Table 1.

### Bariatric surgery reverses obesity-related pathological changes in the monocyte compartment

**Fig. 3:**
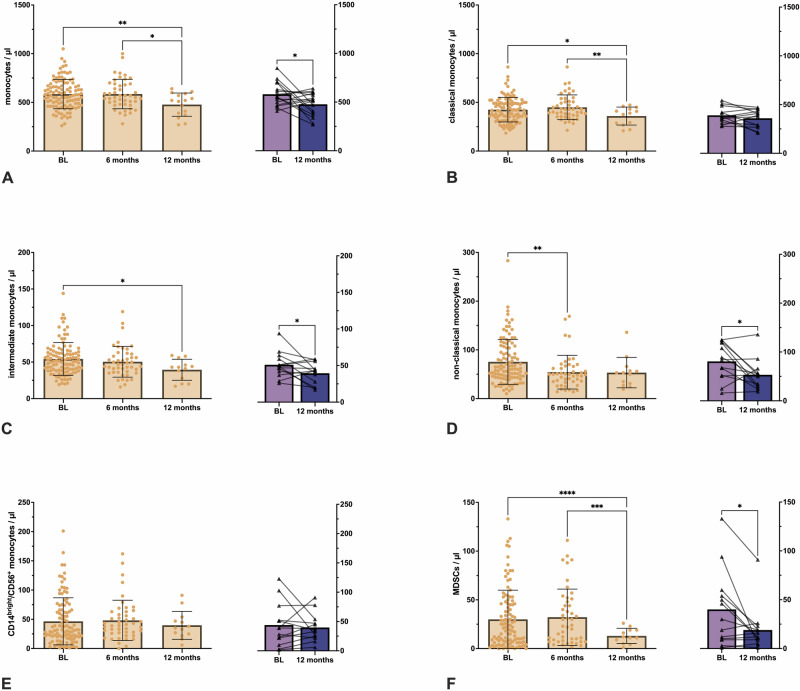
Quantification of the longitudinal changes of distinct monocytic subpopulations during the study period. **A** Overall monocyte count in people with obesity (yellow bars) at baseline (*n* = 111), six months (*n* = 46) and twelve months (*n* = 16). Scatter plots with bar show mean and standard deviation (SD). The purple and blue bars on the right depict a paired analysis comparing n = 13 patients (baseline vs. month 12) instead of a group comparison. **B–F** Numbers of classical monocytes, intermediate monocytes, non-classical monocytes, CD14^bright^/CD56^+^ monocytes, and M-MDSCs at the depicted time points. Each dot represents one patient. Significances as indicated. ^*^ – *p* < 0.05, ^**^ – *p* < 0.01, ^***^ – *p* < 0.001, ^****^ – *p* < 0.0001; *CD* cluster of differentiation, *M-MDSC* monocytic myeloid-derived suppressor cells.

The absolute monocyte count decreased after the surgical procedure. Peripheral monocyte counts fell considerably during the second half of the observation period and were significantly lower after twelve months both compared to baseline (*p* = 0.0035) as well as compared to the six months timepoint (*p* = 0.0063). Analysis did not reveal significant change between baseline and month six though (Fig. 3A). The specific type of bariatric surgery had no effect on monocyte populations.

More detailed analyses of the monocyte subpopulations (see Supplementary Fig. 1 for gating strategies) revealed significant changes throughout the monocyte compartment.

Six months after bariatric surgery, classical monocyte counts were not significantly reduced compared to baseline (Fig. 3B). However, a drop occurred again between month six and month twelve, resulting in significantly lower frequencies after twelve months compared to both baseline and the six-month time point (*p* = 0.0332 comparing baseline vs. month twelve and *p* = 0.0088 for month six vs. month twelve).

As for intermediate monocytes, a significant reduction was seen between baseline and month twelve (*p* = 0.0239, Fig. 3C).

Non-classical monocytes declined more rapidly during the initial six months of observation (*p* = 0.0035), but their decrease did not continue further after twelve months (Fig. 3D). The paired analysis of all patients that were evaluated at baseline and month twelve, revealed an additional difference between baseline and month 12 (*p* = 0.0299).

Regarding CD14^bright^/CD56^+^ monocytes, only a non-significant trend towards decreasing frequencies was seen (*p* > 0.1 for all comparisons, Fig. 3E).

As shown in Fig. 3F, Monocytic myeloid-derived suppressor cells (M-MDSCs) decreased significantly between baseline and month twelve (*p* < 0.0001), but also between month six and month twelve (*p* = 0.0006).

The paired analysis for the overall monocyte counts and each subpopulation comparing baseline and month twelve (Fig. 3*, each on the right*) emphasizes the robustness of our data regardless the dropout rate.

**Fig. 4:**
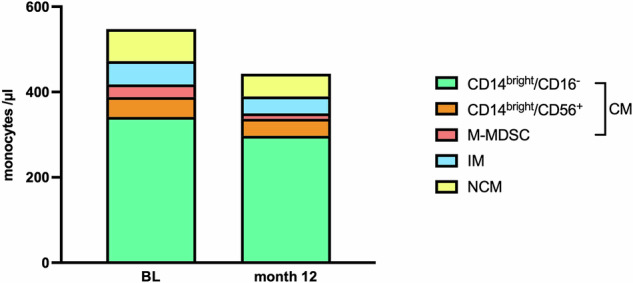
Reversion of monocyte compartment perturbation after bariatric surgery. *BL* baseline, *CD* cluster of differentiation, *CM* classical monocytes, *IM* intermediate monocytes, *M-MDSC* monocytic myeloid-derived suppressor cells, *NCM* non-classical monocytes.

Since we have shown the expansion of classical monocytes in people with obesity before [31], the finding of an overall decreasing number of the classical monocytic compartment after bariatric surgery is striking and further illustrated in Fig. 4.

**Fig. 5:**
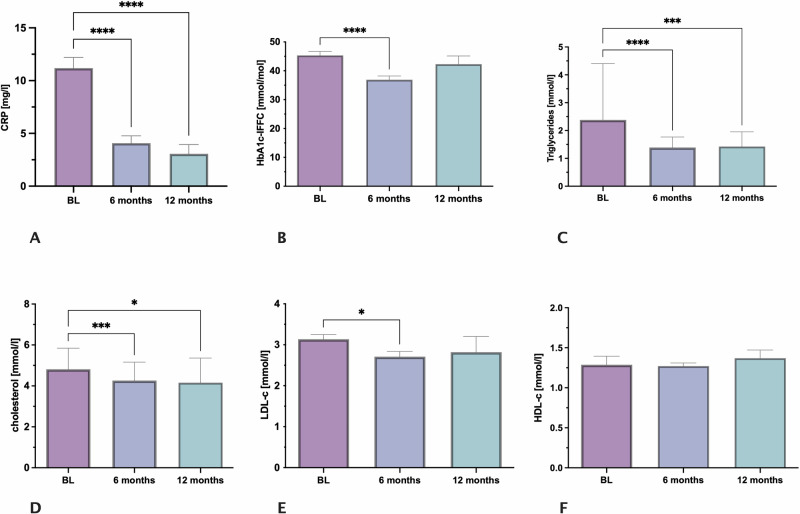
Changes in parameters of inflammation, glucose and lipid metabolism from baseline to six and twelve months after bariatric surgery. **A–F** The bars depict the variables at three different time points: baseline, after 6 months, after 12 months. Shown are mean and standard error of the mean (SEM). Significances as indicated. ^*^ – *p* < 0.05, ^***^ – *p* < 0.001, ^****^ – *p* < 0.0001; *BL* baseline, *BMI* body mass index, *CRP* C-reactive protein, *HDL* high-density lipoprotein, *HbA1c* glycosylated hemoglobin, *LDL* low-density lipoprotein.

In accordance with these findings, laboratory parameters of inflammation and both lipid and carbohydrate metabolism changed after bariatric surgery (Fig. 5). A significant decrease of CRP levels at six and twelve months after surgery (*p* < 0.0001 for both timepoints, Fig. 5A) was observed. In addition, glycosylated hemoglobin (HbA1c) was significantly decreased after six and twelve months in response to bariatric surgery (*p* < 0.0001 and 0.0474, respectively, Fig. 5B). Triglycerides also declined significantly after surgery (*p* < 0.0001 and 0.0177, respectively, Fig. 5C) and so did total cholesterol (*p* = 0.0005 and 0.0380, respectively, Fig. 5C).

Of interest, LDL-cholesterol showed a significant decrease after six (p = 0.0158), but not after twelve months (Fig. 5E). Although we did not observe a significant increase of HDL-cholesterol, we found a rising trend at both, six and twelve months after baseline (*p* = 0.0662 and 0.0977, respectively – Fig. 5F). As shown in Table 1, the LDL/HDL ratio, a surrogate marker of dyslipidemia [33], declined significantly at both months six and twelve (*p* = 0.0045 and 0.0343, respectively).

### Quantitative expression analysis of surface markers

More detailed flowcytometric analysis also revealed phenotypic changes in the three monocyte subpopulations, which are differentiated according to the intensity of the detectable surface expression of CD14 and CD16.

The distinction between classical, intermediate, and non-classical monocytes is determined by the downregulation of CD14. In addition, stimulation of monocytes with cytokines like tumor necrosis factor (TNF) and interferon γ (IFNγ) in vitro has also been shown to reduce CD14 expression [34, 35], which acts as a co-receptor for the detection of bacterial lipopolysaccharide. We hypothesized, therefore, that reduced monocyte activation due to attenuation of adipose tissue inflammation might also decrease CD14 expression and analyzed mean fluorescent intensity of CD14 staining in the subpopulations longitudinally.

**Fig. 6:**
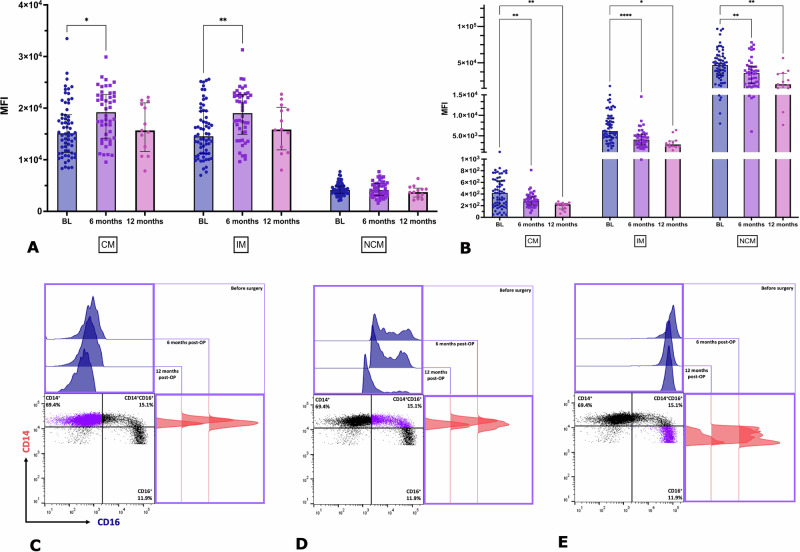
Changes in monocyte surface marker expression during the study period. **A** Expression of CD14 (mean fluorescence intensity [MFI]) in classical, intermediate and non-classical monocytes at baseline, after six months and after twelve months. **B** CD16 expression in classical, intermediate and non-classical monocytes at baseline, after six months and after twelve months. Depicted are median and interquartile range (IQR). Each dot represents one patient. **C–E** Longitudinal expression of CD14 and CD16 in one exemplary individual in classical **C**, intermediate **D** and non-classical **E** monocytes. ^*^ – *p* < 0.05, ^***^ – *p* < 0.001, ^****^ – *p* < 0.0001; *CD* cluster of differentiation.

Expression of CD14 increased in both, classical and intermediate monocytes within 6 months after bariatric surgery (*p* = 0.0272 and 0.0087, respectively, Fig. 6A). No increase of CD14 expression was detected among non-classical monocytes, which, by definition, exhibit very low or absent CD14 levels.

Conversely, the expression of the FcγRIII (CD16), which in general characterizes an enhanced proinflammatory phenotype in monocytes, decreased in all subpopulations at all time points, with the most pronounced decrease occurring in intermediate monocytes after 6 months (*p* < 0.0001, Fig. 6B).

Longitudinal surface marker expression after bariatric surgery in an exemplary individual is further illustrated in Fig. 6C–E.

## Discussion

Our study confirms an expansion of monocytes in obesity, likely underlying the accumulation of macrophages in adipose tissue. Classical and intermediate monocytes, characterized by higher CCR2 expression [7], show increased adipose tissue migration. Bariatric surgery markedly reduced peripheral monocyte counts—particularly within specific subpopulations—along with improvements in body fat, metabolic, and inflammatory parameters.

The best characterized alteration of the monocyte compartment in autoimmune and chronic inflammatory diseases is the consistent increase of intermediate monocytes. Intermediate monocytes have recently been investigated in more detail and were found to increase in healthy aging [28], in particular in CMV positive individuals [27], and were confirmed to be associated with the activity of various autoimmune diseases including SLE [26], adult-onset Still’s disease [23], Takayasu arteritis [22], Behçet’s disease [21], anti-neutrophil cytoplasmatic antibody-associated vasculitis [17], and RA-associated interstitial lung disease [18]. Cardiovascular disease studies have shown intermediate monocytes to also be pathogenetically relevant in atherosclerosis [14–16] and CAD [13, 36, 37], in particular in patients with concurrent T2DM [12]. Intermediate monocytes’ associations with diverse inflammatory diseases highlight their therapeutic potential, though targeted elimination has shown limited success to date. In RA, their frequencies decrease with effective anti-TNF therapy [38]; in SLE, type I interferon blockade yields similar reductions [39].

A Dutch study examined the 18-month effects of combined lifestyle intervention (CLI) on monocyte subsets in obesity, encompassing diet, exercise, and cognitive behavioral therapy [40]. Despite modest reductions in weight (108.4 to 103.2 kg), fat mass (49.2 to 45.4 kg), and BMI (37.4 to 35.8 kg/m²), neither total nor subpopulation monocyte counts declined [40]. This contrasts with the profound monocyte reductions in our study, suggesting substantial adipose tissue loss is required to reverse obesity-driven myelopoiesis. Notably, CLI decreased CD14, CD36, CD45, and CD64 (classical/intermediate monocytes) and CD16 (non-classical/intermediate) expression [40].

Recently, it was demonstrated that very-low-calorie ketogenic diet is able to reduce frequencies of intermediate and non-classical monocytes to some extend [41], although the decrease was limited and no influence on classical monocytes could be detected.

In view of those results, the profound normalization of nearly all monocyte subpopulations, and particularly the significant reduction of intermediate monocytes, is of special interest, and most likely caused by the profound therapeutic efficacy of bariatric surgery. Quantitative surface marker analysis demonstrated a uniform reduction in CD16 surface expression across all monocyte subsets, accompanied by an increase in CD14 expression in classical and intermediate monocytes after six months. This phenotypic shift indicates a reversion of intermediate monocytes toward the less pro-inflammatory classical phenotype. Moreover, weight loss is associated with decreased circulating pro-inflammatory cytokines and a relative increase in anti-inflammatory mediators such as IL-10, which is known to induce CD14 re-expression [42].

A recent publication investigating people with obesity before and six months after Roux-en-Y gastric bypass surgery revealed that, while inflammatory markers and leukocyte numbers were decreasing to levels observed in healthy lean subjects, there was still a residual functional and transcriptional hyperinflammatory monocyte phenotype after six months [43]. Further investigations will have to determine whether this remaining hyperinflammatory state diminishes over a longer follow-up or in dependence of the cause of weight loss (different surgery methods, dietary interventions, use of Glucagon-like Peptide-1 receptor agonists).

Taselaar et al. [44] showed that morbid obesity triggers inflammaging-like shifts across adaptive (T and B cells) and innate (NK cells) immune compartments, with elevated counts compared to lean controls. Bariatric surgery largely normalized these lymphocyte and NK cell subsets by 12–18 months postoperatively, though some declined below control levels; similarly, elevated classical and intermediate monocyte subsets (but not non-classical) normalized by twelve months.

The marked reduction of M-MDSCs after bariatric surgery in our cohort is noteworthy. This monocytic population, typically expanded under chronic inflammation, suppresses innate and adaptive immunity [45]. In cancer, M-MDSCs accumulate in the tumour microenvironment, where they inhibit T and NK cell activity and promote resistance to antitumour therapies [46]. Their immunosuppressive effects are mediated by nitric oxide, IL-10, and immune checkpoints such as PD-L1 [46]. In obesity, M-MDSC expansion [31] may counteract pro-inflammatory monocytes and could contribute to the elevated cancer risk [47–49], though protective metabolic effects have also been described [50]. The postoperative decline in M-MDSCs observed here may therefore reduce cancer risk. Moreover, M-MDSC levels correlated with BMI and body fat at baseline, and most strongly with CRP six months post-surgery.

Frequencies of CD14^bright^/CD56^+^ monocytes did not change significantly after bariatric surgery. This pro-inflammatory monocyte subpopulation was found to be expanded in Crohn’s disease, rheumatoid arthritis and immunosenescence [29, 51, 52], and to be hyperinflammatory and strongly increased in severe COVID-19 cases [53]. We demonstrated previously that individuals with obesity have an increased proportion of CD14^bright^/CD56^+^ monocytes [31]. The absence of a significant decrease after bariatric surgery may reflect persistent inflammatory activity, an insufficient follow-up period, or the impact of patient loss during follow-up.

Following bariatric surgery, numbers of non-classical monocytes also significantly decreased within six months and remained on that lower level after twelve months. Remarkably, non-classical monocytes showed a strong relationship to CRP levels six and twelve months after the intervention. Although non-classical monocytes are primarily involved in the resolution of inflammation, they have also been linked to progression of chronic autoimmune and inflammatory diseases [54]. They are also known to accumulate in the elderly, reflecting senescent cells that are involved in inflammaging [55]. In obesity, non-classical monocytes were reported to be expanded before [56–59]. High-intensity interval training in adults with obesity over an 8-week period resulted in restoration of the CD16^+^ monocyte balance by reducing the non-classical monocytes [60]. Similarly, our study also showed that non-classical monocytes, despite their apparent contribution to monocytosis in obesity, seem to be successfully normalized after bariatric surgery.

Notably, we did not find increased numbers of non-classical monocytes in obesity compared to lean controls before. We did see increased non-classical monocytes in obesity and coincidental T2DM though [31]. Insulin resistance in obesity is the combined consequence of altered functions of insulin target cells and the accumulation of macrophages secreting pro-inflammatory mediators [2]. Half the patients (49.5%) that participated in this study had a diagnosed T2DM, further 9.9% had a known impaired glucose tolerance. This proportion of patients is comparable to our previous investigation [31]. Although this might not explain our finding of increased numbers of non-classical monocytes in obesity and their decrease after bariatric surgery, numbers of non-classical monocytes were particularly high in patients with T2DM at baseline. This in turn suggests a contribution of non-classical monocytes in the inflammatory condition that encourages the development of an insulin resistance and ultimately T2DM.

### Study limitations

The primary limitation of this study is its single-centre design. Additionally, a substantial proportion of participants were lost to follow-up, particularly at the 6-month post-bariatric surgery assessment. Notwithstanding these constraints, the consistent results offer meaningful insights into obesity-associated immunological alterations. Furthermore, COVID-19 pandemic-related factors like lockdowns and vaccinations may confound monocyte subpopulation changes post-bariatric surgery, as lockdowns enhanced monocyte-derived cytokine production and innate immune responsiveness, while vaccinations dampened it. These effects in people living with HIV and healthy cohorts highlight the need to stratify future studies by pandemic exposure [61].

## Conclusions

Most importantly, we were able to demonstrate that both, the monocytosis as well as the perturbation of the monocyte compartment in individuals with obesity can be successfully reversed by bariatric surgery and the subsequent weight loss. This conclusion is of tremendous importance since reversing these changes not only reduces the number of monocytes with an inflammatory phenotype such as intermediate monocytes, but also immunosuppressive monocytes that might contribute to the increased rate of malignancies in obesity.

Following this normalization, the chronic inflammatory condition in people with obesity might be attenuated or even eliminated. Furthermore, the increased cancer risk associated with obesity could be reduced.

## Supplementary information

FACS Gating Strategy

Supplementary Table 1

## Data Availability

The data underlying this article will be shared on reasonable request to the corresponding author.
