# Supplementary material for: Bariatric surgery and subsequent weight loss lead to a reversal of peripheral blood monocytosis in obesity
Source: Int J Obes (Lond). 2026 Apr 24;50(8):1742–50. doi: 10.1038/s41366-026-02093-4 (PMC13461359; doi:10.1038/s41366-026-02093-4)
Supplement: Supplementary file 1 [file 41366_2026_2093_MOESM2_ESM.docx]

**Supplementary table 1**

Correlation between monocyte subsets (absolute count) and variables of obesity, inflammation, glucose/lipid metabolism at baseline,

6 and 12 months. Depending on data distribution, Pearson correlation coefficients or Spearman's rank correlation coefficients was calculated.

|  | monocytes | | CM | | IM | | NCM | | CD14^bright^/CD56^+^ | | M-MDSC | |
| --- | --- | --- | --- | --- | --- | --- | --- | --- | --- | --- | --- | --- |
|  | *r* | *p* | *r* | *p* | *r* | *p* | *r* | *p* | *r* | *p* | *r* | *p* |
| baseline | | | | | | | | | | | | |
| BMI | **0.211** | **0.031** | 0.154 | 0.118 | **0.225** | **0.022** | **0.270** | **0.006** | 0.097 | 0.329 | **0.203** | **0.037** |
| fat mass | 0.198 | 0.067 | 0.168 | 0.125 | 0.157 | 0.152 | **0.229** | **0.035** | 0.155 | 0.155 | **0.248** | **0.021** |
| CRP | **0.198** | **0.039** | **0.193** | **0.045** | **0.199** | **0.039** | 0.125 | 0.196 | 0.091 | 0.347 | 0.180 | 0.061 |
| Cholesterol | -0.173 | 0.072 | -0.033 | 0.736 | -0.053 | 0.585 | -0.098 | 0.311 | 0.087 | 0.221 | 0.043 | 0.344 |
| LDL-C | -0.070 | 0.476 | 0.006 | 0.950 | 0.086 | 0.376 | -0.039 | 0.688 | 0.058 | 0.232 | -0.012 | 0.259 |
| HDL-C | -0.007 | 0.946 | 0.041 | 0.675 | -0.056 | 0.563 | 0.039 | 0.691 | 0.084 | 0.571 | -0.181 | 0.718 |
| Triglycerides | 0.040 | 0.677 | 0.017 | 0.857 | 0.048 | 0.622 | 0.062 | 0.524 | 0.158 | 0.042 | 0.206 | 0.064 |
| HbA1c | **0.206** | **0.033** | 0.128 | 0.191 | 0.047 | 0.635 | 0.177 | 0.069 | 0.183 | 0.876 | 0.393 | 0.650 |
| 6 months | | | | | | | | | | | | |
| BMI | 0.137 | 0.376 | 0.085 | 0.581 | 0.111 | 0.471 | 0.151 | 0.327 | 0.100 | 0.520 | 0.140 | 0.366 |
| fat mass | 0.058 | 0.768 | 0.023 | 0.907 | 0.047 | 0.813 | 0.095 | 0.630 | 0.135 | 0.494 | 0.332 | 0.084 |
| CRP | **0.394** | **0.007** | **0.307** | **0.038** | 0.127 | 0.398 | **0.404** | **0.005** | 0.089 | 0.556 | **0.350** | **0.017** |
| Cholesterol | -0.208 | 0.166 | -0.146 | 0.333 | -0.232 | 0.121 | -0.176 | 0.243 | -0.226 | 0.132 | -0.046 | 0.759 |
| LDL-C | -0.155 | 0.303 | -0.087 | 0.565 | -0.196 | 0.192 | -0.177 | 0.239 | -0.227 | 0.129 | -0.048 | 0.750 |
| HDL-C | -0.280 | 0.060 | -0.261 | 0.080 | -0.072 | 0.632 | -0.131 | 0.387 | 0.009 | 0.952 | 0.018 | 0.903 |
| Triglycerides | -0.093 | 0.538 | -0.049 | 0.746 | -0.076 | 0.614 | -0.161 | 0.284 | -0.123 | 0.415 | 0.076 | 0.616 |
| HbA1c | **0.315** | **0.035** | 0.272 | 0.070 | 0.208 | 0.169 | 0.086 | 0.574 | -0.041 | 0.791 | 0.197 | 0.194 |
| 12 months | | | | | | | | | | | | |
| BMI | **0.526** | **0.025** | 0.467 | 0.126 | 0.448 | 0.145 | 0.494 | 0.103 | 0.197 | 0.539 | 0.511 | 0.089 |
| fat mass | -0.581 | 0.419 | 0.140 | 0.860 | -0.456 | 0.544 | **-0.981** | **0.019** | -0.350 | 0.650 | -0.852 | 0.148 |
| CRP | 0.426 | 0.078 | 0.152 | 0.637 | 0.340 | 0.280 | **0.688** | **0.013** | 0.020 | 0.952 | 0.521 | 0.082 |
| Cholesterol | 0.197 | 0.433 | 0.500 | 0.098 | 0.437 | 0.155 | **0.579** | **0.049** | 0.454 | 0.138 | 0.478 | 0.116 |
| LDL-C | 0.298 | 0.230 | 0.512 | 0.089 | 0.516 | 0.086 | **0.672** | **0.017** | 0.400 | 0.198 | 0.577 | 0.050 |
| HDL-C | -0.439 | 0.068 | 0.010 | 0.975 | -0.248 | 0.438 | -0.374 | 0.231 | 0.266 | 0.404 | -0.444 | 0.148 |
| Triglycerides | 0.288 | 0.246 | 0.309 | 0.329 | 0.462 | 0.131 | 0.350 | 0.264 | 0.232 | 0.468 | 0.359 | 0.251 |
| HbA1c | 0.059 | 0.811 | -0.098 | 0.749 | 0.113 | 0.714 | 0.230 | 0.449 | -0.008 | 0.980 | 0.514 | 0.072 |

BMI – body mass index; CM – classical monocytes; CRP – C-reactive protein; HDL – high-density lipoprotein; IM – intermediate monocytes;

LDL – low-density lipoprotein; M-MDSC – monocytic myeloid-derived suppressor cells; NCM – non-classical monocytes; WHR – waist-hip ratio
